# Variation of National and International Guidelines on Respiratory Protection for Health Care Professionals During the COVID-19 Pandemic

**DOI:** 10.1001/jamanetworkopen.2021.19257

**Published:** 2021-08-04

**Authors:** Gabriel Birgand, Nico T. Mutters, Jonathan Otter, Vanessa M. Eichel, Didier Lepelletier, Daniel J. Morgan, Jean-Christophe Lucet

**Affiliations:** 1 National Institute for Health Research Health Protection Research Unit in Healthcare Associated Infections and Antimicrobial Resistance at Imperial College London, London, United Kingdom; 2 Regional Center for Infection Prevention and Control Pays de la Loire, Centre Hospitalier Universitaire de Nantes, Nantes, France; 3 Institute for Hygiene and Public Health, University Hospital Bonn, Bonn, Germany; 4 Centre for Infectious Diseases, Heidelberg University Hospital, Heidelberg, Germany; 5 Infection Control Unit, Centre Hospitalier Universitaire de Nantes, Nantes, France; 6 University of Maryland School of Medicine, Baltimore; 7 VA Maryland Healthcare System, Baltimore; 8 Infection Antimicrobials Modelling Evolution, French Institute for Medical Research, University Paris Diderot, Sorbonne Paris Cité, Paris, France; 9 Infection Control Unit, Hôpital Bichat-Claude Bernard, Assistance Publique-Hôpitaux de Paris, Paris, France

## Abstract

This systematic review assesses variation in international and national guidelines on respiratory protection for health care professionals during the COVID-19 pandemic.

## Introduction

Conflicting evidence surrounding SARS-CoV-2 transmission, particularly airborne transmission, may have contributed to heterogeneous recommendations for respiratory protection across countries and organizations.^1^ Variability among guidelines may generate confusion, anxiety, and mistrust among health care professionals (HCPs) regarding the ability of respiratory protection to prevent SARS-CoV-2 transmission. We assessed variation in international and national guidelines on respiratory protection for HCPs in hospital settings during the first year of the COVID-19 pandemic.

## Methods

The Nantes University Hospital determined that this systematic review was exempt from formal review by the institutional review board and from written informed consent because this study used publicly accessible documents. We followed the Preferred Reporting Items for Systematic Reviews and Meta-analyses (PRISMA) reporting guideline. Infection prevention and control guidelines on respiratory protection for HCPs published from January 1 to December 31, 2020, by leading organizations in 4 countries and 2 international organizations were collected through a monthly consultation of their official websites (eMethods in the Supplement). We defined HCPs as all workers employed by a hospital. For each included guideline, we extracted data regarding the organization, date of publication, type of respiratory protection recommended (ie, medical face mask [MF] or respirator, including N95 or N99 or filtering facepiece [FFP2 or FFP3]), indication for use, and lists of aerosol-generating procedures (AGPs). We classified the indications for respirators as recommended during AGPs, recommended for targeted continuous use in high-risk areas, and recommended during contact with individuals with suspected or confirmed infections in any circumstance. The indications for MFs were categorized as universal face masking recommended, targeted continuous face masking recommended, or recommended during contact with individuals with suspected or confirmed infections only. Targeted continuous use was defined as the wearing of a respirator or MF by all HCPs during their entire shifts in clinical areas with patients with or without COVID-19. Universal face masking was defined as a requirement for all HCPs entering the facility to wear an MF. Data were analyzed from December 2020 to March 2021 using Excel version 2010 (Microsoft).

## Results

Among 114 screened guidelines, 55 guidelines (48.2%) were excluded (24 were excluded because they were not national or international infection prevention and control recommendations, 14 because they were for nonhospital settings, and 17 because the guideline versions were updated on topics other than respiratory protection) and 59 guidelines (51.8%) were included. In January 2020, all initial guidelines recommended respirators (ie, N95 or N99 or FFP2 or FFP3) for HCPs in direct contact with patients with suspected or confirmed COVID-19 infection (Figure). After February 10, 2020, the European Centre for Disease Prevention and Control (ECDC) introduced the recommendation for use of MFs by HCPs caring for patients with COVID-19 in the absence of available respirators, followed 1 month later by the same recommendation from the US Centers for Disease Control and Prevention (CDC), while respirators (ie, FFP3 and N99) were a strict recommendation for AGPs by the ECDC and CDC. The World Health Organization (WHO) was the first reviewed entity to strictly recommend MFs for HCPs in contact with patients with suspected or confirmed COVID-19 infection, limiting the use of respirators to during AGPs. France and the UK followed WHO guidance 1 week later by issuing new versions of their guidelines. Germany was the first country to recommend universal face masking for HCPs at entry to a hospital, doing so on March 22, 2020. This recommendation appeared later in other countries: April 13 for the US CDC, May 6 for France, and August 20 for the UK. Overall, 13 different guidelines detailed what was an AGP, providing lists varying from 3 to 14 procedures. Intubation and bronchoscopy were the most frequently cited AGPs, with intubation appearing in 13 guideline updates and bronchoscopy in 12 guideline updates (Table).

**Figure.**
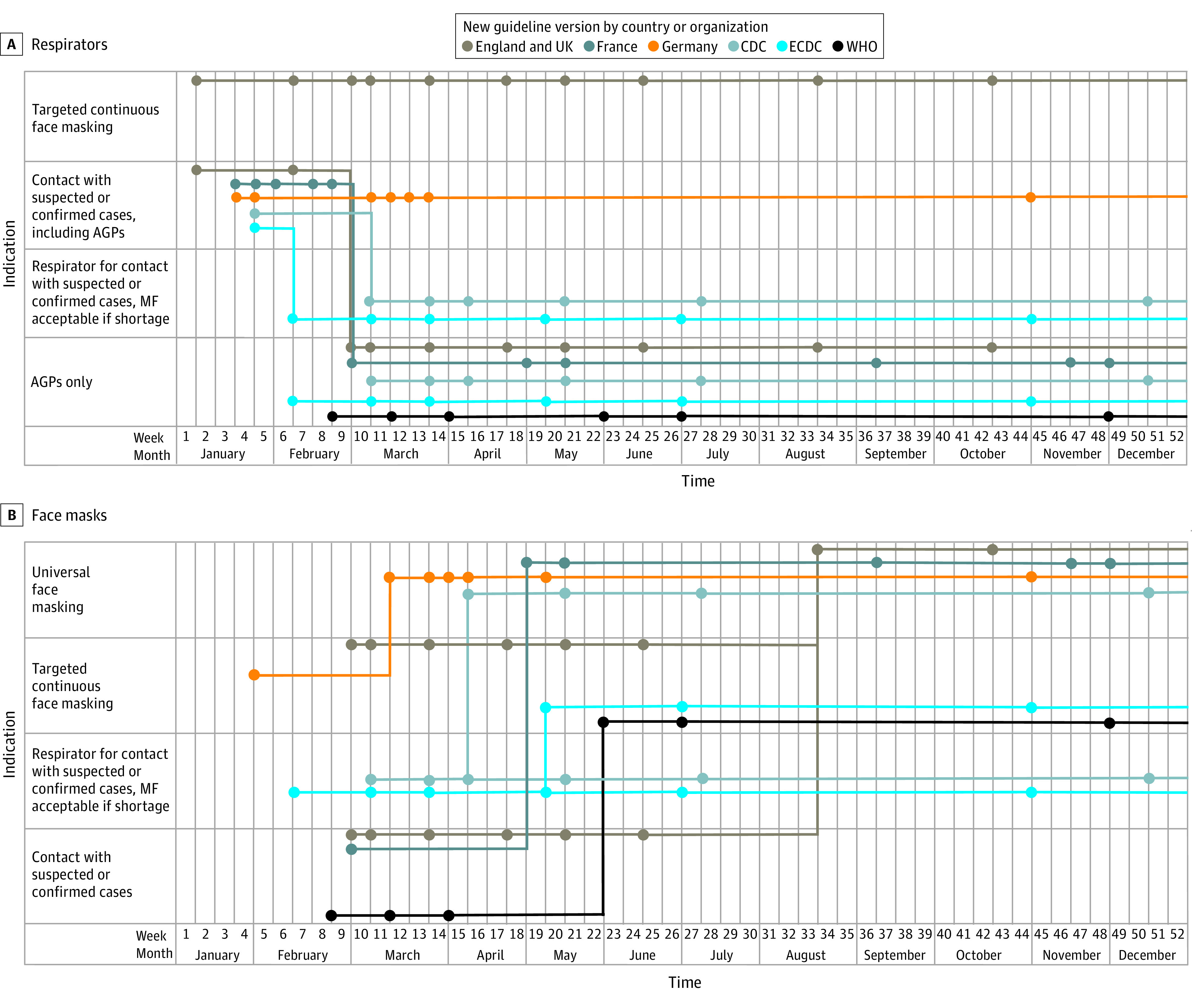
Evolution of Recommendations for Respiratory Protection Among Health Care Professionals by Country and Organization AGP indicates aerosol-generating procedure; CDC, US Centers for Disease Control and Prevention; ECDC, European Centre for Disease Prevention and Control; MF, medical face mask; WHO, World Health Organization.

**Table.** Description of the Aerosol-Generating Procedures Listed in National and International Guidelines

Procedure	Procedure listed in guideline												
	England and UK: PHE, PHA-NI, PHW, HPS, NHS		France: HCSP		Germany: RKI	Germany: DGKH	US: CDC		Europe: ECDC			World: WHO	
	Mar 6^a^	May 17	Mar 10	Nov 20	Jan 26	Jan 31	Mar 10	Apr 5	Mar 13	Mar 31	Oct 6	Feb 27	Dec 1
Intubation	Yes	Yes	Yes	Yes	Yes	Yes	Yes	Yes	Yes	Yes	Yes	Yes	Yes
Extubation	Yes	Yes	Yes	Yes	No	No	No	Yes	No	No	No	No	No
Manual ventilation	Yes	Yes	No	No	No	Yes	Yes	Yes	No	Yes	Yes	Yes	Yes
Suctioning	Yes	Yes	Yes	Yes	No	No	Yes	Yes	No	Yes	Yes	No	No
Tracheotomy and tracheostomy	Yes	Yes	No	No	No	Yes	No	No	No	Yes	Yes	Yes	Yes
Bronchoscopy	Yes	Yes	Yes	Yes	Yes	Yes	No	Yes	Yes	Yes	Yes	Yes	Yes
Dental procedures	Yes	Yes	Yes	Yes	Yes	No	No	No	No	No	No	No	Yes
NIV	Yes	Yes	Yes	Yes	No	Yes	Yes	Yes	No	Yes	Yes	Yes	Yes
High-frequency oscillating ventilation	Yes	Yes	No	No	No	No	No	No	No	No	Yes	No	No
HFNO (also called high-flow nasal cannula)	Yes	Yes	No	Yes	No	No	No	Yes	No	No	Yes	No	No
Induction of sputum	Yes	Yes	Yes	Yes	No	No	No	Yes	Yes	No	Yes	No	Yes
Upper ENT airway procedures	No	Yes	No	No	No	No	No	No	No	No	No	No	No
Upper GI endoscopy	No	Yes	No	No	No	No	No	No	No	No	No	No	No
Surgical and postmortem procedures	No	Yes	Yes	Yes	No	No	No	No	No	No	No	No	Yes
Aerosol therapy	No	No	Yes	Yes	No	No	Yes	Yes	No	Yes	No	No	No
Nasopharyngeal sample	No	No	Yes	Yes	No	No	No	No	No	No	No	No	No
Respiratory functional exploration	No	No	Yes	Yes	No	No	No	No	No	No	No	No	No
Mechanical ventilation	No	No	Yes	Yes	No	Yes	No	No	No	Yes	No	No	No
Cardiopulmonary resuscitation	No	No	No	No	No	Yes	Yes	Yes	No	Yes	Yes	Yes	Yes
Physical prone positioning	No	No	No	No	No	No	No	No	No	Yes	No	No	No

Abbreviations: CDC, Centers for Disease Control and Prevention; DGKH, Deutsche Gesellschaft für Krankenhaushygiene; ECDC, European Centre for Disease Prevention and Control; ENT, ear, nose, and throat; GI, gastrointestinal; HCSP, Haut Conseil de la Santé Publique; HFNO, high-flow nasal oxygen; HPS, Health Protection Scotland; NHS, National Health Services; NIV, noninvasive ventilation; PHE, Public Health England; PHA-NI: Public Health Agency Northern Ireland; PHW, Public Health Wales; RKI, Robert Koch Institute; WHO, World Health Organization.

^a^ All dates were in 2020.

## Discussion

In this systematic review, we observed 4 strategies regarding the use of respirators: (1) systematic: recommended for care of COVID-19 patients, as in German guidelines; (2) flexible: recommended with use of MFs in the absence of available respirators, as in US and ECDC guidelines; (3) unit based, as in UK guidelines; and (4) recommended exclusively during AGPs, as in French and WHO guidelines. These discrepancies may reflect controversies related to SARS-CoV-2 transmission routes.^2,3^ The most recent assessment of the clinical evidence for the risk of transmission of acute respiratory infections to HCPs caring for patients undergoing AGPs dates back to a 2012 systematic review of a limited volume of studies (ie, 10 nonrandomized studies, including 5 relevant case-control studies and 5 retrospective cohort studies).^4^ More evidence is still needed to clearly define what AGPs are and the level of risk associated with different procedures.^5^

Initial uncertainties surrounding asymptomatic transmission of SARS-CoV-2 and the initial global shortage of MFs may explain some of the early delays in universal face-masking recommendations. The growing recognition of the role played by asymptomatic HCPs and the publication of a major positioning paper may have triggered the extension of face masking.^6^ Some guideline updates may have been dated incorrectly, representing the main study limitation.

Over the course of the COVID-19 pandemic, contextual factors (eg, resource limitations) may have generated variability in recommendations owing to a lack of scientific evidence. Inconsistencies in respiratory protection guidelines between neighboring countries created confusion over optimal measures. Strong collaborations between national and international organizations are critical in such circumstances.

## Notes

Supplement. eMethods.
