# Supplementary material for: Variation of National and International Guidelines on Respiratory Protection for Health Care Professionals During the COVID-19 Pandemic
Source: JAMA Netw Open. 2021 Aug 4;4(8):e2119257. doi: 10.1001/jamanetworkopen.2021.19257 (PMC8339937; doi:10.1001/jamanetworkopen.2021.19257)
Supplement: Supplement. — eMethods. [file jamanetwopen-e2119257-s001.pdf]

Supplemental Online Content

Birgand G, Mutters NT, Otter J, et al. Variation of national and international guidelines on respiratory protection for health care professionals during the COVID-19 pandemic. *JAMA Netw Open*. 2021;4(8):e2119257. doi:10.1001/jamanetworkopen.2021.19257

eMethods.

This supplemental material has been provided by the authors to give readers additional information about their work.

© 2021 Birgand G et al. *JAMA Network Open.*

eMethods.

Methods adopted for the systematic review of guidelines.

This study was conducted in accordance with the Preferred Reporting Items for Systematic Reviews and Meta-Analyses (PRISMA) guidelines.

Search strategy

The leading organizations were chosen by consensus between the study investigators. We performed a monthly systematic search for all guidelines on respiratory protection dedicated to HCP working in hospital facilities, published between the 1 January to the 31 December 2020 on the official websites of leading organizations in four countries and two international organizations. The reference lists of the identified guidelines were scanned to derive any further relevant guidelines. The last search was run to 5 January 2021. (Supplementary Table 1) The literature search was performed by two authors working independently of each other. (Supplementary Table 2)

Inclusion and exclusion criteria

We included infection prevention and control guidelines for healthcare professionals in hospital settings during the COVID-19 pandemic providing recommendations on respiratory protections, published in English, French and German between 1 January 2020 and 31 December 2020. Only new guidelines versions, and updated versions with specific changes in the recommendations on respiratory protections were included. We excluded guidelines for the community, primary care, hemodialysis, dental, nursing homes settings, guideline-related documents (leaflets, checklist, frequently asked questions), and guidelines versions updated on other topics than respiratory protections.

© 2021 Birgand G et al. *JAMA Network Open.*

Guidelines selection and data extraction

Two reviewers performed the monthly systematic search on the official websites of leading organizations, and screened all titles and full texts. Both reviewers were infection control specialists, and at least one of them was involved in COVID-19 prevention and control in her/his country at the national level. (Supplementary Table 2) Two reviewers, with an international expertise, independently reviewed the titles and content of the international guidelines, and disagreements were resolved by a third person (GB or JCL). For each included guideline, the following information were extracted: the organization or society publishing the guideline, the date of publication, the type of respiratory protection recommended (medical facemask [MF] or respirator [N95/99, Filtering Face Piece FFP2/3]), indication for use, and lists of aerosol generating procedures (AGP).

Data analysis

We conducted a descriptive analysis of the indications for respirators and MF of the included guidelines. We described the variation and evolution of indications according to countries/organizations and according to time in weeks. We classified the indications for respirators in: targeted continuous use in high-risk areas, contact with suspected or confirmed cases in any circumstance, and during AGP. The indications for MF were categorized in: targeted continuous face masking, contact with suspected or confirmed cases only, and universal face masking. Targeted continuous use was defined as wearing a respirator or MF by all HCP during their entire shift in clinical areas with COVID-19 or non-COVID-19 patients. Universal face masking was defined as a requirement to wear a MF by all HCP entering the facility.

© 2021 Birgand G et al. *JAMA Network Open.*

Supplementary Table 1. Leading organizations selected for the study, and their official websites searched.

No	Organization	URL
1	Public Health England	https://www.gov.uk/government/publications/wuhan-novel-coronavirus-infection-prevention-and-control
3	COREB	https://www.coreb.infectiologie.com/fr/covid-19.html
4	French society for infection control	https://www.sf2h.net/publications/coronavirus-2019-ncov
5	High council for public health, Ministry of Health	https://www.hcsp.fr/explore.cgi/avisrapports
6	RKI, Robert Koch institute;	https://www.rki.de/DE/Content/InfAZ/N/Neuartiges_Coronavirus/nCoV_node.html
7	DGKH, Deutsche Gesellschaft für Krankenhaushygiene;	https://www.krankenhaushygiene.de/informationen/fachinformationen/empfehlungen-der-dgkh/
8	DVV, Deutsche Vereinigung zur Bekämpfung der Viruskrankheiten e. V (German Association for fighting viral infections);	https://www.g-f-v.org/node/1232
9	DGHM, Deutsche Gesellschaft für Hygiene und Mikrobiologie (German Society for Hygiene and Microbiology);	https://www.dghm.org/informationen-zum-neuartigen-corona-virus/
10	Centre for Diseases prevention and Control	https://www.cdc.gov/coronavirus/2019-ncov/hcp/infection-control-recommendations.html
11	European Center for Diseases prevention and Control	https://www.ecdc.europa.eu/en/all-topics-z/coronavirus/threats-and-outbreaks/covid-19/prevention-and-control/patient-and-case
12	World Health Organization	https://www.who.int/publications/i

© 2021 Birgand G et al. *JAMA Network Open.*

Supplementary Table 2: Reviewers information's.

Country	Reviewers	Degree	Specialty	Position	Level of the position
France	Gabriel Birgand,	PharmD, PhD	Infection control	Head of the regional center for infection control Pays de la Loire, Nantes, France	Regional, National
	Didier Lepelletier	MD, PhD	Infection control	Head of the infection control unit, Nantes University hospital, France<br>Vice-president Haut Conseil de Santé Publique France	Hospital, National
	Jean-Christophe Lucet	MD, PhD	Infection control	Head of the infection control unit, Bichat-Claude Bernard hospital, Paris, France	Hospital, National
UK	Jonathan Otter	PhD	Infection control	General manager ICP at Imperial College Healthcare NHS Trust in London, and an Honorary Senior Lecturer in the National Institute for Health Research Health Protection Research Unit (NIHR HPRU) in Healthcare Associated Infections and Antimicrobial Resistance at Imperial College London	Hospital, National
	Gabriel Birgand	PharmD, PhD	Research	Research associate in the National Institute for Health Research Health Protection Research Unit (NIHR HPRU) in Healthcare Associated Infections and Antimicrobial Resistance at Imperial College London	International
Germany	Nico T. Mutters	MD, MPH	Infection control	Head of Infection Control Heidelberg, Baden-Württemberg, Germany	Hospital, National
	Vanessa M. Eichel	MD	Infection control	Infection Control Heidelberg, Baden-Württemberg, Germany	Hospital
USA	Daniel J. Morgan	MD, MSc	Epidemiology and public health	Infectious disease physician and hospital epidemiologist at the University of Maryland School of Medicine and VA Maryland Healthcare System, USA	Hospital, National
	Gabriel Birgand	PharmD, PhD	Infection control	None in USA	
ECDC, WHO	Gabriel Birgand,	PharmD, PhD	Infection control	Head of the regional center for infection control Pays de la Loire, Nantes, France	Regional, National
	Jean-Christophe Lucet	MD, PhD	Infection control	Head of the infection control unit, Bichat-Claude Bernard hospital, Paris, France	Hospital, National

© 2021 Birgand G et al. *JAMA Network Open.*
